# In‐Plane Combination of Micropillars with Distinct Aspect Ratios to Resist Overload‐Induced Adhesion Failure

**DOI:** 10.1002/advs.202400972

**Published:** 2024-05-08

**Authors:** Dongwu Li, Ruozhang Li, Kangbo Yuan, Ao Chen, Ning Guo, Chao Xu, Wenming Zhang

**Affiliations:** ^1^ School of Astronautics Northwestern Polytechnical University Xi'an 710072 China; ^2^ State Key Laboratory of Mechanical System and Vibration School of Mechanical Engineering Shanghai Jiao Tong University Shanghai 200240 China; ^3^ School of Mechanics Civil Engineering and Architecture Northwestern Polytechnical University Xi'an 710072 China

**Keywords:** adhesion failure, bioinspired adhesion, micropillars, pressure‐sensitive adhesion, space docking capture

## Abstract

Bioinspired micropillar adhesives have shown broad application prospects in space capture and docking, due to their strong adhesion, good environmental adaptability, and reusability. However, when performing space missions, unavoidable contact collision with target objects may cause large deformation of the micropillars, resulting in the loss of adhesion ability. This study reports a novel micropillar adhesive through the in‐plane combination of micropillars (IPCM) with different aspect ratios, consisting of small pillars for retaining strong adhesion and large ones for resisting overload‐induced adhesion failure. It is demonstrated that the IPCM array can still maintain 85% of the adhesion peak after static large deformation compared to a general micropillar array composed of the same pillars. The impact of element size and layout of the IPCM, as well as detachment velocity on adhesion performance under high preload is discussed. Furthermore, finite element contact analysis qualitatively reproduces the experimentally observed micropillar deformations and attributes the overload‐induced adhesion failure to the redistribution of surface normal stress. Finally, the potential application of the IPCM in dynamic capture is demonstrated on different objects. The proposed IPCM opens up new design concepts for practical applications of bioinspired adhesives in space capture and docking.

## Graphical abstract

A novel micropillar adhesive consisting of small pillars for retaining strong adhesion and large pillars for resisting overload‐induced adhesion failure is developed. It is demonstrated that the developed in‐plane combination of micropillars (IPCM) array can maintain 85% of the adhesion peak after large deformation compared to conventional micropillar arrays. Good adhesion stability under high load enables it to cope with low‐speed impacts during space capture and docking.

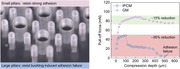

## 1 Introduction

Biomimetic fibrillar adhesives are a functional surface material whose design is inspired by the powerful adhesion performance of the pads of animals such as geckos and beetles, which have sophisticated hierarchical microstructures. Such structured adhesives, mainly relying on intermolecular van der Waals forces to achieve adhesion,^[^ ^1^ ^]^ have shown broad application prospects in biomedical devices,^[^ ^2^, ^3^ ^]^ climbing robots,^[^ ^4^, ^5^ ^]^ pick‐and‐place operations,^[^ ^6^, ^7^, ^8^ ^]^ and space manipulation,^[^ ^9^, ^10^, ^11^ ^]^ due to their excellent adhesion, switchability, environmental adaptability, and reusability. In particular, physical or chemical modifications enable bioinspired adhesives to adapt to extreme environments in space.^[^ ^9^ ^]^ Therefore, they can be integrated into the end effector of space robots to execute complex space missions such as orbital debris capture, docking, asteroid orbital transfer, etc.

Over the past two decades, versatile bioinspired adhesives have been developed, mainly in terms of tip geometry, switchability, environmental adaptability, and sensing. Both the shape and size of the microfibril tip have a significant impact on its adhesion.^[^ ^12^, ^13^, ^14^ ^]^ Numerous studies have shown that mushroom‐shaped microstructures have better adhesion performance due to the mechanism of central crack nucleation and the elimination of stress singularity at the contact edges.^[^ ^15^, ^16^, ^17^, ^18^ ^]^ Of course, new tip geometry‐dominated microstructures are still constantly emerging.^[^ ^19^, ^20^ ^]^ The application of bioinspired adhesives in pick‐and‐place operations requires good adhesive switchability, i.e., quick and efficient attachment and detachment. Achieving switchable adhesion often relies on external stimuli such as mechanical forces, light, and electric and magnetic fields.^[^ ^21^, ^22^, ^23^, ^24^, ^25^, ^26^, ^27^ ^]^ Isla and Kroner^[^ ^21^ ^]^ proposed a two‐step switchable micropillar adhesive with two distinct heights, which can exhibit three adhesion states by applying different levels of preload. This adhesive utilizes the elastic instability of micropillars induced by compression overload to produce a nonadhesive state, thereby achieving load‐controlled adhesion switching. Essentially, such design regulates adhesion performance by varying the actual contact area. Li et al.^[^ ^22^ ^]^ designed a composite mushroom‐shaped adhesives consisting of a pillar structure layer, a stiffness‐modulating thermoplastic polyurethane layer, and an electrothermal film. The adhesion state is switched through voltage‐controlled adjustable structural stiffness. However, the advantage of this switchability can only be reflected in the pick‐and‐place to nonflat objects. For more research on switchable adhesion, please refer to the review literature.^[^ ^23^, ^24^ ^]^ In terms of environmental adaptability, researchers focused on the effects of humidity, temperature, and target surface roughness on fibrillar adhesion.^[^ ^30^, ^31^, ^32^, ^33^ ^]^ A humid environment will remarkably reduce intermolecular interactions of adhesives, which can be improved by mimicking the structures of a mussel or tree frog.^[^ ^30^ ^]^ The temperature effect is mainly reflected in the influence on material modulus, i.e., the changes within the range of glassy and rubbery states. In addition, developing fibrillar adhesives with sensing functions for practical application scenarios has gradually become an emerging direction in this field.^[^ ^34^, ^35^, ^36^, ^37^ ^]^

**Figure 1:**
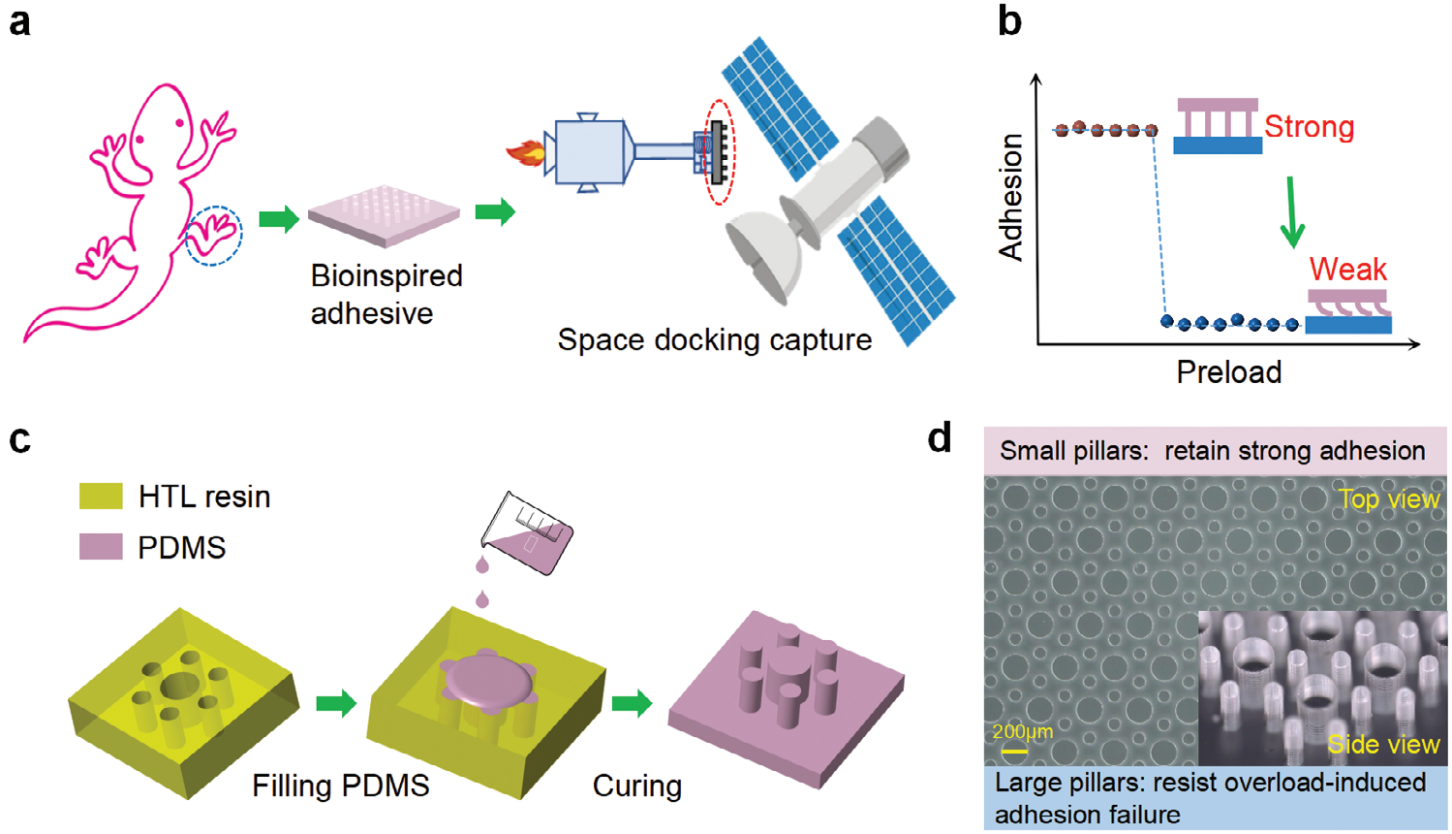
a) Schematic diagram of the application of bioinspired adhesives in space docking capture, b) pressure‐sensitive effect of micropillar adhesion: high preload will lead to a significant reduction in adhesion or even failure. c) illustration of the fabrication process of IPCM arrays, d) optical microscopic photograph, and basic design principles of IPCM arrays.

Different from the above designs, structured adhesives encounter harsh environmental challenges when extended to space missions, including high/low‐temperature cycling, radiation, atomic oxygen, vacuum, ultraviolet (UV) exposure, and inevitable low‐speed impact collisions.^[^ ^9^, ^38^ ^]^ Seong et al.^[^ ^39^ ^]^ reported a nanocomposite adhesive made of polydimethylsiloxane (PDMS) reinforced with multi‐walled carbon nanotubes of different concentrations, which exhibited excellent thermal stability and adhesion performances. However, its adhesion stability in space low‐temperature environments remains unclear. Xia et al.^[^ ^40^ ^]^ developed a low‐temperature reversible microfibrillar adhesive fabricated by phenyl‐containing PDMS elastomers, which is non‐crystallizable with excellent low‐temperature elasticity, and can well maintain adhesion strength even at −120 °C. However, comprehensive consideration of the adaptability of micropillar adhesives at space high (sunward side) and low (nightside) temperatures is still a challenging issue. Henrey et al.^[^ ^41^ ^]^ experimentally evaluated the effect of ambient pressure on the adhesion of mushroom‐shaped adhesives, which showed scarcely loss of adhesion compared to samples tested at room temperature and atmospheric pressure. Day et al.^[^ ^42^ ^]^ investigated the effect of gamma irradiation on the adhesion performance of PDMS elastomer‐based adhesives. They found that high‐dose gamma irradiation resulted in a 55% reduction in adhesion performance and permanent deformation of microstructures. Nevertheless, it is unclear to what extent radiation plays a part in this deformation. Bareth et al.^[^ ^43^ ^]^ demonstrated that long‐term exposures to oxygen plasma significantly affect the performance of silicone‐based dry adhesives. They recommended rapid deployment of adhesives before capturing space targets to minimize this surface damage. In addition to environmental factors, when capturing non‐cooperative targets in space, the end effector will inevitably collide with the target surface at low speeds. Such a collision may cause large deformation and even buckling of surface micropillars. (**Figure** **1**a) Extensive experiments have shown that micropillar buckling can lead to adhesion failure,^[^ ^7^, ^44^, ^45^, ^46^ ^]^ as shown in Figure 1b. Therefore, developing micropillar adhesives that can resist such failure is of great significance for their application in space capture. This is a challenging task arising from the mutual exclusivity of the structural stability (to resist large deformation) against the mechanical compliance (to form intimate contact) of fibrillar structures.^[^ ^47^ ^]^ Despite much research into the pressure‐sensitive behavior of fibrillar adhesives, there is still a lack of methods to improve adhesion stability and reliability under the premise of successful adhesion, which is of greater concern in space missions.

In this study, to avoid the possible instability of the micropillars and further adhesion failure caused by collision in space applications, we develop a novel micropillar adhesive through the in‐plane combination of micropillars (IPCM) array with different aspect ratios, which consists of small pillars for retaining adhesion and large pillars for resisting overload‐induced adhesion failure. It is experimentally demonstrated that the developed IPCM arrays maintain good adhesion despite large deformations (buckling and sliding) of micropillars. Furthermore, we reveal the mechanical mechanism of the overload‐induced adhesion failure through experiments and finite element contact analysis. Demonstration experiments for dynamically capturing different objects are also given.

## 2 Results and Discussion

### 2.1 Fabrication of IPCM Array

The IPCM samples made of PDMS were prepared using the molding method, as shown in Figure 1c. Photosensitive high‐temperature lamination (HTL) resin molds with hexagonally arranged microholes were prepared based on Projection Micro Stereolithography technology. The PDMS was filled into the HTL resin molds under vacuum, and a thin piece of glass was covered on the PDMS to form a flat backing layer. Subsequently, the IPCM samples were obtained after curing and demolding. Figure 1d presents photographs of the fabricated IPCM array (a regular hexagon with a side length of 3 mm, Figure S1 (Supporting Information), which consists of 384 small pillars (100 µm diameter and aspect ratio. λ_*l*_ = 3) and 169 large pillars (200 µm diameter and aspect ratio. λ_*l*_ = 1.5). Each large pillar is surrounded by six small pillars, which are distributed in a regular hexagon with a side length of 200 µm.

### 2.2 Adhesion Performance of the IPCM Arrays

A custom‐made adhesion test setup was used to characterize the adhesion of IPCM arrays (Experimental Section and Figure S2, Supporting Information). A plano‐convex lens with a diameter of 83.335 mm was adopted as a probe, and alignment was achieved by manually adjusting a positioning stage based on real‐time visual images of probe‐micropillars contact. Using the displacement‐controlled testing method, we performed loading‐pause‐unloading testing (Section 4.3) on micropillar arrays over a wide range of compression depths until the probe made contact with the backing layer outside the edge of micropillar arrays. To demonstrate the adhesion stability of the IPCM array under high preload, a general micropillar (GM) array with all micropillars having the same size (100 µm diameter and aspect ratio. λ_*l*_ = 3) was set up as a control.

**Figure 2:**
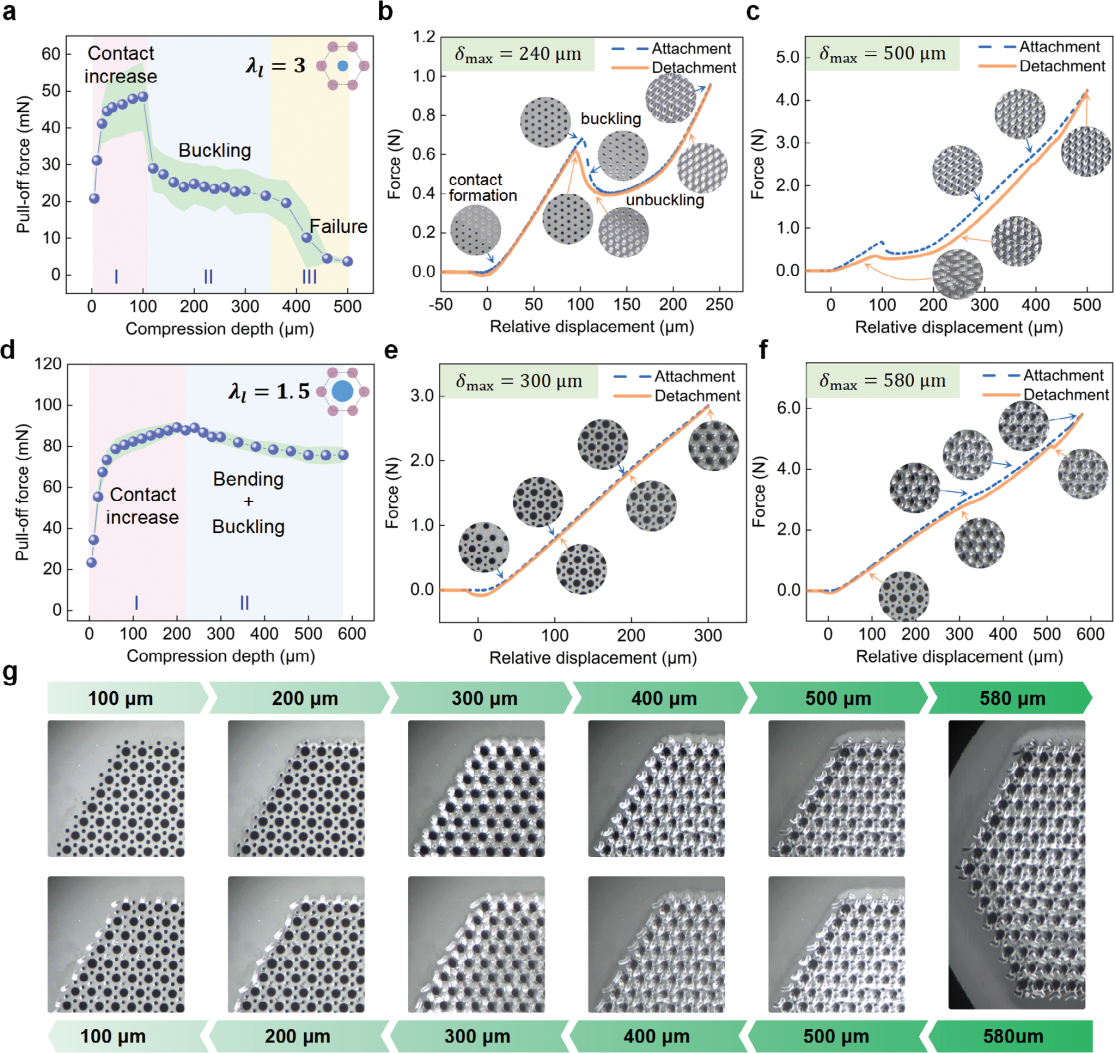
a) Pull‐off force as a function of compression depth for the GM array with an aspect ratio λ_*l*_ of 3, b) force‐displacement curves of the GM array at 240 µm compression depth and snapshots of the contact status of local micropillars at different moments, the dark and light areas represent the contact and detached area, respectively. c) force‐displacement curves of the GM array at 500 µm compression depth, d) pull‐off force as a function of compression depth for the IPCM array consisting of small micropillars with λ_*l*_ = 3 and large micropillars with λ_*l*_ = 1.5, e) force‐displacement curves of the IPCM array at 300 µm compression depth, f) force‐displacement curves of the IPCM array at 580 µm compression depth, g) snapshots of the local area of the IPCM array at different loading/unloading displacements.

**Figure** **2**a shows the measured pull‐off force as a function of compression depth δ_max_ for the GM array, where the green shading represents the error band calculated from the results of multiple tests. It can be seen that there are three stages in which the pull‐off force changes significantly: (I) adhesion increasing (0<δ_max_<100 µm), (II) adhesion decay (100 µm<δ_max_<350 µm), and (III) adhesion failure (350 µm<δ_max_<500 µm). To our knowledge, this three‐stage variation in pull‐off force on micropillar arrays has never been reported.

During stage I, as the compression depth increases, the pull‐off force first increases considerably (when δ_max_<30 µm), and then the increment diminishes (when 30 µm<δ_max_<100 µm). Such a substantial growth in pull‐off force is determined by the nature of non‐planar contact, i.e., an increase in compression depth causes the curved probe to contact more pillars, while the subsequent slight increase in pull‐off force is due to better intimate contact at relatively high loads. Figure S3 (Supporting Information) illustrates the force‐displacement curves during the attachment and retraction process at a compression depth of 100 µm, as well as in‐situ images of the contact area at different moments. It shows that the force‐displacement relation is almost linear when the relative displacement is in the range of 30 to 100 µm, which corroborates the above explanation for the slight increase in adhesion from the side. The in situ images indicate that in this case no visible bending deformation was observed in any micropillars.

During stage II, there is a sharp decline in pull‐off force when the compression depth increases to 120 µm. Subsequently, the pull‐off force maintains a slowly decreasing trend over a wide compression depth range of 120 to 340 µm and finally drops to 50% of the peak value. Referring to Movie S1 (Supporting Information) obtained at a compression depth of 240 µm, it can be observed that the pillars underwent obvious bending deformation. Also, a deformation wave corresponding to the deflection of pillars propagated rapidly from the probe center to the circumference, accompanied by the change from top contact to the side. This large bending deformation should be caused by the buckling of pillars, as shown in Figure 2b, where the force in the attachment stage (blue dashed line) suddenly drops within the interval of relative displacement 105 – 120 µm. The explanation will be detailed in Section 2.5. By the way, that is why we do not adopt the force‐controlled testing method. The inset at 110 µm displacement shows the loss of partial contact area and bending deformation of pillars. As the relative displacement further increases, one side of the deflected pillars comes into contact with the probe, while the other side touches the backing layer (see the inset at 240 µm displacement). Obviously, the contact stiffness (i.e., the slope of the force‐displacement curve) at this time is greater than the linear contact stiffness before buckling. During the retraction of the probe, the pillars first break out of contact with the backing layer (see the inset at 220 µm displacement), subsequently uncoil, and regain the top contact (see the inset at 95 µm displacement) before complete detachment. Figure 2b shows that the force‐displacement curve during buckling does not coincide with that during unbuckling, indicating the existence of buckling‐induced hysteresis,^[^ ^7^, ^45^ ^]^ which means that some mechanical energy (i.e., the area enclosed by the force‐displacement curve) is dissipated in this process. The mechanism of this dissipation could be buckling‐caused elastic energy release and friction action between the probe and pillars. In addition, it should be emphasized that although the snapshot indicates that the top contact appears to form again, it cannot recover to the intimate contact before buckling. As a result, some adhesion is lost.

At greater compression depths (Stage III in Figure 2a), the pull‐off force further declines significantly (when 350 µm<δ_max_<450 µm), and finally down to ≈3 mN (7% of the peak value) at 500 µm compression depth. Figure 2c plots the force‐displacement curves during the attachment/detachment process, corresponding to Movie S2 (Supporting Information). It can be seen that during attachment, the contact force increases monotonically with increasing compression depth after buckling‐induced reduction. The bent pillars slipped sideways and were pressed tightly against the backing layer under the overload. In addition, some of the pillars also came into contact with each other and even interlock. The compression was stopped until the backing layer outside the pillar area of the sample being tested came into contact with the probe. During retraction, the peripheral probe‐backing layer contact was first broken. Then, under the action of elastic restoring force, the pillars attempted to overcome the adhesion between its side and the backing layer and the interlocking of adjacent pillars, and to return to the initial good contact status with the probe but failed. Movie S2 (Supporting Information) shows that merely a few pillars came in contact with the probe again, but only partially.

In contrast, the developed IPCM array exhibits good adhesion maintenance capability, with no adhesion failure occurring even at high compression depths, except for a small amount of adhesion loss, as shown in Figure 2d. When the compression depth δ_max_<220 µm (Stage I), the pull‐off force growth trend of the IPCM array is the same as that of the GM array. However, the difference is that at larger compression depths (such as 200 µm), the pillars at the outer edge of the IPCM array tilt first (Movie S3, Supporting Information). In particular, the tilted pillars are oriented toward the contact center, which is opposite to the deformation direction of the GM array. Meanwhile, we can also observe a deformation mismatch between the array and the backing layer, which is caused by the difference in stiffness between the two. Although the outer pillars of the IPCM array undergo visible tilt deformation at this time, thus losing part of the contact area, it can be seen from Movie S3 (Supporting Information) that the lost contact will be formed again during the unloading process. Therefore, there is no adhesion attenuation when the compression depth δ_max_<220 µm.

As the compression depth further increases, the pull‐off force decreases to a certain extent. After the compression depth is greater than 500 µm, it remains almost stable (Figure 2d). Figure 2e depicts the contact force as a function of the relative displacement. δ_max_ = 300 µm. It shows that when the relative displacement is larger than 220 µm, there is a slight drop in the contact stiffness (i.e., the slope of the force‐displacement curve). Moreover, the attachment and detachment curves do not exactly coincide. Movie S4 (Supporting Information) shows during the attachment process, the small pillars are tilted, bent, and buckled in sequence, and the deformation direction is toward the contact center of the probe. We can also observe a deformation wave propagating inward from the periphery of the IPCM array (that is, the outer pillars deformed first). Meantime, the large pillars also tilted to a certain extent. During retraction, the IPCM array followed a reverse deformation process. The large pillars returned to the upright state, while all small pillars regained top contact but lost part of the contact area, which is also the reason for the adhesion decay.

Figure 2f plots the force‐displacement curve at a compression depth of 580 µm, indicating a significant hysteresis phenomenon when the displacement is greater than 320 µm. Besides, the force experienced a slight fluctuation at a displacement of 500 µm, which should be caused by the separation of the probe from the backing layer. The corresponding video (Movie S5, Supporting Information) shows that the larger compressive load caused the buckled small pillars to stretch, possibly with twisting, and eventually lie flat on the backing layer (Figure 2g captured at 100 µm intervals from Movie S5, Supporting Information). The deflection of the large pillars can also be observed. During the unloading process, all large pillars formed top contact again, while small pillars lost some contact area, as shown in Figure 2g. The above results demonstrate the excellent adhesion stability of the developed IPCM array, which can still maintain 85% of the adhesion peak even under high compression loads. Next, we will investigate the influence of the layout and geometrical dimensions of large pillars in IPCM arrays on their adhesion performance and attempt to seek the working principle of IPCM arrays.

### 2.3 Effects of Micropillar Array Layout and Geometric Dimensions

**Figure 3:**
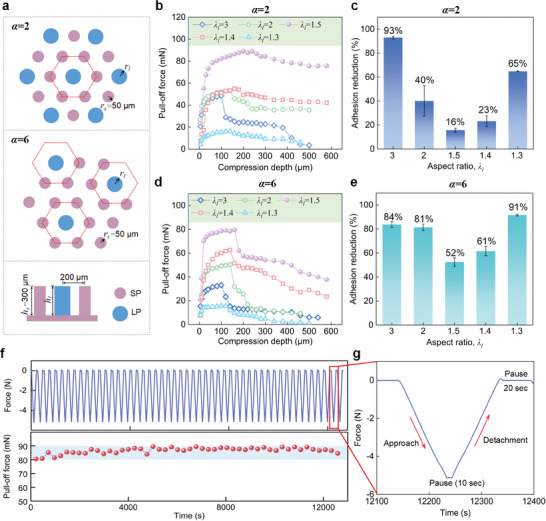
a) Two hexagonal layouts of IPCM array in which the height of small pillars is 300 µm, the ratio of the number of small pillars to large ones is 2 for the first layout and that for the second layout is 6, b) pull‐off force as a function of compression depth for different IPCM arrays (the first layout *α* =  2) in which the aspect ratio λ_*l*_ of large micropillars is 3, 2, 1.5, 1.4, and 1.3, c) adhesion reduction (the ratio of the difference between the pull‐off force at the maximum compression depth and the maximum pull‐force to the maximum pull‐off force) for different IPCM arrays (the first layout *α* =  2), d) pull‐off force as a function of compression depth for different IPCM arrays (the second layout *α* =  6), e) adhesion reduction for different IPCM arrays (the second layout *α* =  6), f) Measured force and extracted pull‐off force as a function of time, g) force overtime measured during a single cycle.

IPCM arrays with different layouts were prepared, as shown in **Figure** **3**a, where all small pillars have the same height of 300 µm and diameter of 100 µm. Each large pillar is surrounded by 6 small pillars, arranged in a regular hexagon with a side length (center‐to‐center) of 200 µm. The first layout (the upper one in Figure 3a) includes 553 pillars and the ratio of the number of large pillars to small pillars *α* =  2, while the second layout (the bottom one) has 657 pillars and *α* =  6. In each layout, we considered five micropillar arrays with large pillars of different aspect ratios: λ_*l*_ = 3 (100 µm diameter and 300 µm height), λ_*l*_ = 2 (150 µm diameter and 300 µm height), λ_*l*_ = 1.5 (200 µm diameter and 300 µm height), λ_*l*_ = 1.4 (200 µm diameter and 280 µm height), and λ_*l*_ = 1.3 (200 µm diameter and 260 µm height).

Figure 3b illustrates the measured pull‐off force as a function of the compression depth for the arrays with the first layout (*α* =  2). In the previous section, we have discussed in detail the variation pattern of the pull‐off force of the micropillar arrays (for the cases of.λ_*l*_ = 3 and 1.5), which will not be further elaborated on here. The evolution of the pull‐off force with increasing compression depth for the arrays with λ_*l*_ = 2 and 1.4 is similar to that for the array with λ_*l*_ = 1.5. It increases first, then decays, and finally stabilizes. The peaks in both cases are very close, but the final attenuation is different. The in situ monitoring video displays that almost all the buckled small pillars in the array (λ_*l*_ = 2) did not form the top contact with the probe again during retraction. Therefore, its adhesion loss is significant. For the array (λ_*l*_ = 1.4), although the in situ monitoring shows that the two differently shaped pillars detached from contact with the probe almost simultaneously, the elastic restoring force of small pillars offset part of the adhesion of large pillars due to the height difference (20 µm) between the small and large pillars. Therefore, although its nominal contact area is the same as that of the array (λ_*l*_ = 1.5), overall, its adhesion is lower than that of the latter. In the case of.λ_*l*_ = 1.3, the pull‐off force first rises to its peak and then slowly decays. Within the entire compression depth range, the pull‐off force is at a relatively low level, below 20 mN. This is due to the relatively large height difference (40 µm) between the small and large pillars, which can be verified from the surveillance video showing that large pillars first detached from contact. Under high compression depths, the adhesion of the arrays (λ_*l*_ = 2, 1.5, and 1.4) can be ultimately maintained at a certain level, while for the cases of.λ_*l*_ = 3 and 1.3, it almost fails.

Figure 3c presents the adhesion reduction of these five arrays. Adhesion reduction is defined as the ratio of the difference between the peak pull‐off force and the pull‐off force at the maximum compression depth to the peak pull‐off force. The smaller the adhesion attenuation, the more stable the adhesion is under overload. The statistical results indicate that the IPCM array (λ_*l*_ = 1.5) has the smallest adhesion reduction, ≈16%, while the adhesion reduction of the arrays (λ_*l*_ = 3 and 1.3) exceeds 60%. Theoretically, a pillar with the same diameter but a smaller height would be more rigid (and thus provide better support). However, this will result in a loss of some adhesion despite a relatively low adhesion reduction (such as the case.λ_*l*_ = 1.4).

The evolution of pull‐off force with increasing compression depth for the arrays with the second layout (*α* =  6) is shown in Figure 3d. All tested micropillar arrays with different sizes exhibited similar evolutionary trends in pull‐off force. It first rapidly increased to a high level within the compression depth range of 20 µm and then continued to rise, but the increment gradually slowed down. After reaching its peak, the pull‐off force first declined significantly and then maintained a slow reduction trend. Except for the arrays (λ_*l*_ = 1.4 and 1.5), the adhesion of the remaining arrays dropped to very low levels at the maximum compression depth, which can be considered as adhesion failure. In situ monitoring showed that under high compressive loads, almost all the small pillars in the above five arrays failed to regain top contact during the unbuckling process. The adhesion reduction of the above five arrays is given in Figure 3e, where the attenuation of all arrays exceeds 50%. Therefore, it can be concluded that since there are relatively few large pillars in the second arrangement (*α* =  6), the overall stiffness of the array area is not high, so the large pillars do not play a role in the deformation recovery of the small pillars. Figure 3f plots measured force and extracted pull‐off force as a function of time for the IPCM array (λ_*l*_ = 1.5) under a compressive depth of 500 µm. It includes 50 cycles with a 20‐s pause after each cycle to allow for stress relaxation (as shown in Figure 3g). The coefficient of variation of the pull‐off force (i.e., the ratio of standard deviation to mean) is less than 2.5%, indicating that the adhesion test has good stability.

Through the above comparison, we can find that both the element layout and the dimension of large pillars will seriously affect the adhesion performance of IPCM arrays. It is worth emphasizing that the design idea of IPCM arrays proposed in this paper is not limited to the above‐mentioned adhesive design. For example, we can also prevent overload‐induced failure and improve adhesion stability through the non‐uniform design of micropillars or even the use of composite materials. In addition, it should be noted that it is unrealistic to blindly increase the diameter of large pillars. This will take up too much space and affect the overall adhesion performance.

### 2.4 Does Detachment Velocity Make a Difference?

Whether the micropillars can form intimate top contact again after sliding and/or buckling under high preloads plays an important role in the adhesion of the micropillar arrays. During unloading, detachment proceeds as a peeling wave. Array adhesion is favored if contact formation on the pillar top surfaces is completed before the peeling wave arrives, but not vice versa. Based on this, we speculate that the detachment velocity may have an impact on the adhesion performance of micropillar arrays under high loads. In other words, the smaller the detachment velocity, the more conducive it is to forming contact again. To answer this question, we study the relation between pull‐off force and compression depth at different detachment velocities in this section. Furthermore, note that micropillar arrays have a rate‐dependent adhesion enhancement effect due to the viscoelastic characteristics of PDMS material.^[^ ^48^, ^49^, ^50^, ^51^ ^]^

**Figure 4:**
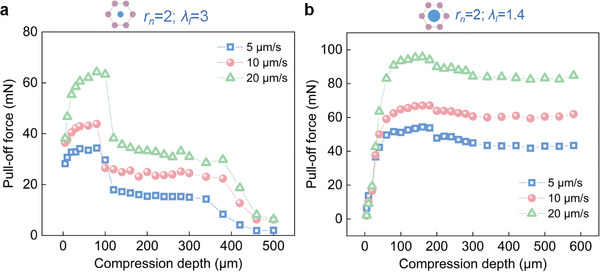
Pull‐off force as a function of compression depth at different detachment velocities: a) for the general micropillar array (*α* =  2, λ_*l*_ = 3), b) for the IPCM array (*α* =  2,.λ_*l*_ = 1.4).

**Figure** **4** plots the pull‐off force with increasing compression depth for the GM array and the IPCM array (*α* =  2 and.λ_*l*_ = 1.4) at different detachment velocities. Unfortunately, we did not observe the adhesion decay with increasing detachment velocity as speculated above. Both sets of curves show that within a given compression depth range, the higher the detachment velocity, the greater the pull‐off force. Given the possible competition between viscoelastic rate‐dependent adhesion and reduced adhesion due to contact loss, it is difficult to conclude that the latter plays a role.

Figure S4 (Supporting Information) shows force‐time plots at compression depths (40, 160, 320, and 480 µm) and higher detachment velocities (50, 100, and up to 1000 µm s^−1^). For the case of 480 µm compression depth and 1000 µm s^−1^ velocity, there are two peaks in the force‐time curve. According to the in situ monitoring, the first peak is caused by the detachment of the probe from the backing layer, while the second is due to the micropillar array detachment. Figure S4e (Supporting Information) compares the pull‐off forces at different detachment velocities, and the variation is consistent with the results in Figure 4a. Since it is difficult to quantify the contribution of rate‐dependent effects to adhesion from experimental results, it cannot be determined whether the detachment velocity affects the contact re‐formation and thus array adhesion. We can only conclude that the rate‐dependent adhesion enhancement due to material viscoelasticity remains effective even after micropillar buckling and/or sliding.

### 2.5 Mechanical Mechanism of Adhesion Decay

To further explore the mechanism of adhesion decay under high preload, we performed a finite element analysis (FEA) on the attachment and detachment of a single pillar. The detailed FEA can be found in Section 4.4, where a bilinear cohesive zone model is used to simulate the adhesive interaction of the probe‐pillar contact, and the material is assumed to be elastic (Viscoelasticity is not considered due to static loading), as shown in Figure S5 (Supporting Information). The loading‐unloading process of the probe on the pillar was simulated. Figure S6 (Supporting Information) qualitatively compares the contact images (captured in tests) of a single pillar with the contact contour obtained by FEA, confirming the feasibility of the analysis method.

**Figure 5:**
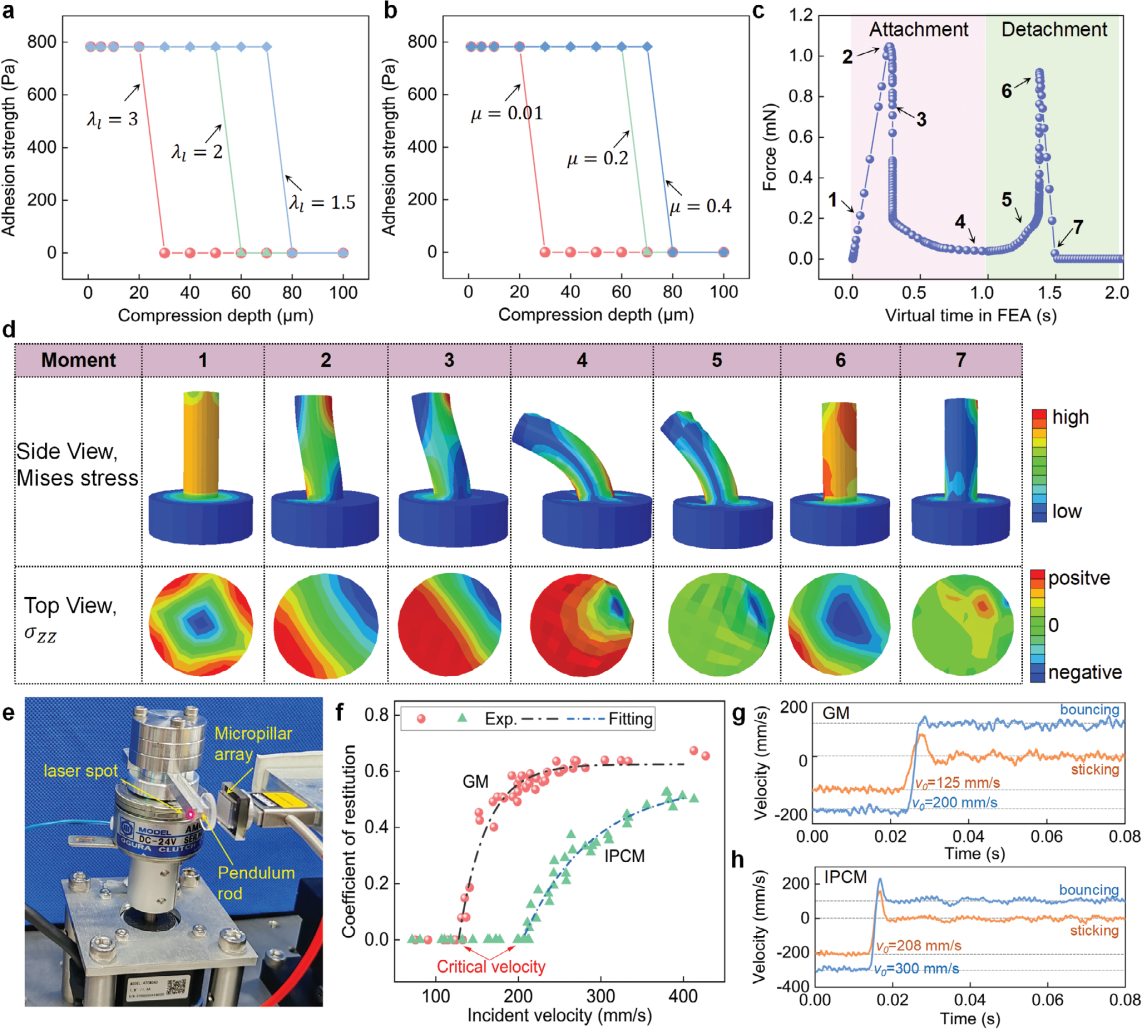
a) Finite element simulated adhesion strength as a function of compression depth for different micropillars with aspect ratios of 3, 2, and 1.5, b) simulated adhesion strength as a function of compression depth at different friction coefficients of 0.01, 0.2, and 0.4, c) simulated force versus virtual time in finite element analysis in the case of friction coefficient *µ* = 0 and compression depth of 80 µm, d) finite element simulated deformation and normal stress distribution of micropillar at different attachment/detachment moments. e) Photograph of the test setup simulating dynamic capture. A pendulum rod can be manually controlled to collide with the bio‐inspired adhesive at a certain initial speed. The speed of the rod and contact force are measured. f) The coefficient of restitution (defined as the ratio of rebound velocity to incident velocity) as a function of incident velocity for GM and IPCM arrays. g) Velocity‐time curves for two typical motion modes during GM array collision. h) Velocity‐time curves for two typical motion modes during IPCM array collision.

**Figure** **5**a illustrates the evolution of adhesion strength with increasing compression depth for pillars with different aspect ratios (λ_*l*_ = 3, 2, and 1.5) but the same height. In this case, the friction coefficient of the probe‐pillar contact is set to be *µ* = 0.01 (for almost an ideal smooth surface). Adhesion strength is defined as the pull‐off force divided by the nominal contact area. The results indicate that the adhesion strength of the three pillars jumps to zero under a certain compression depth, completely losing the adhesion. Moreover, the smaller the aspect ratio, the greater the compression depth at the “jump”. Figure 5b compares the adhesion strength of the pillar (λ_*l*_ = 3) for different friction coefficients (*µ* = 0.01, 0.2, and 0.4) as a function of compression depth, showing that a high friction coefficient results in a larger critical compression depth (corresponding to the onset of adhesion failure). A high friction coefficient will prevent relative sliding between the contact surfaces, thus ensuring intimate contact under a larger compression depth.

Figure 5c plots the contact force of the pillar (λ_*l*_ = 3) as a function of virtual time (in FEA) at a compression depth of 80 µm. Similar to the experimental results, both exhibit force “jump” and loading‐unloading asymmetry. The deformation diagrams of the pillar at different moments are listed in Figure 5d (second row), where the pillar slips sideways and gradually loses top contact during loading, then appears to return to the upright state during unloading. There is no clear evidence that buckling occurred during this process. As a result, in this case, sliding should be the main cause of the force jump. As a comparison, from the deformation animation of the pillar at friction coefficient *µ* = 0.4 (Movie S6, Supporting Information), we can observe that its deformation process involves buckling and sliding of the pillar and that buckling occurs before sliding. The Euler‐Bernouli buckling theory^[^ ^52^ ^]^ is also employed to provide theoretical insights. The critical buckling load *F_cr_* is expressed as *F_cr_* = *n* ^2^ π^2^ *EI*/*h* ^2^, in which *E* is the Young's modulus of the pillar, *I* is the second moment of area and *h* the height of the pillar, *n* is the pre‐factor which is related to the bucked shape and takes different values depending on the constraints at both ends of the pillar. The theoretical critical load (4 mN) is close to the simulated force (4.3 mN) at the jump when we take the pre‐factor *n* = 2 (clamped‐clamped). However, the theoretical values for the very low friction coefficient (*µ* = 0.01) do not match well with the simulated values. This result also proves the existence of buckling instability in the case of relatively high friction coefficients (*µ* = 0.2 and 0.4). Strictly speaking, the dimension of the pillar does not meet the prerequisites of the Euler‐Bernouli buckling theory. Nevertheless, this indirect evidence of buckling is still encouraging.

From the snapshot of the pillar deformation in Figure 5d and the deformation animation (Movie S6, Supporting Information), we can see that the pillar appears to be able to return to its original upright state before surface separation. However, the adhesion still failed. To reveal the adhesion failure mechanism in this case, we obtained the normal stress distribution contour on the top surface of the pillar at different times, as shown in Figure 5d (third row). The results show that at the critical separation point, although surface contact was re‐formed, the surface normal stress underwent a redistribution compared to the initial contact. At this point, the contact is eccentric and there are cracks in most surrounding areas, which is not conducive to adhesion. Combining finite element analysis and experimental observations, it can be found that the sliding and buckling of pillars are the direct causes of adhesion failure, and buckling often occurs before sliding (considering friction on the actual contact surface). Its failure mechanism is normal stress redistribution due to large deformations.

### 2.6 Application of IPCM Array for Dynamic Capture

To demonstrate the advantage of the IPCM array in dynamic capture, we conducted collision tests and compared the dynamic responses of IPCM (*α* =  2, λ_*l*_ = 1.5) and GM arrays. As shown in Figure 5e, a test device simulating dynamic capture was set up, where the micropillar array is connected to a load cell fixed in a manual positioning stage. A pendulum rod connected to a fixed axis can be manually controlled to collide with micropillar arrays at a certain initial velocity (i.e., incident velocity *v_0_*). A spherical probe is pasted on the free end of the pendulum rod and directly contacts micropillar arrays under rotational load. The speed of the free end of the pendulum rod and the contact force between the probe and micropillar array are measured (Experimental Section and Figure S8, Supporting Information).

Figure 5f plots the coefficient of restitution (COR, defined as the ratio of rebound velocity to incident velocity) as a function of incident velocity for IPCM and GM arrays. It should be noted that when the COR equals 0, the pendulum rod adheres to micropillar arrays after collision. On the contrary, it indicates that the pendulum rod is rebounded. The results show that the IPCM array has a higher critical incident velocity (208 mm s^−1^) at which the adhesive can critically capture the pendulum rod than the GM array (125 mm s^−1^). Overall, the COR of the IPCM array in bouncing mode is much lower than that of the GM array at the same incident velocity. Figure 5g,h shows two typical velocity‐time curves of the GM and IPCM arrays under bouncing and sticking modes (Movie S7, Supporting Information). The above dynamic contact tests demonstrate that IPCM arrays have higher adhesion and energy dissipation. In addition, to display the potential of IPCM arrays in space capture and docking, we conducted demonstration experiments on IPCM arrays for capturing three different objects. They include a plano‐convex lens with 83.3 mm diameter, an aluminum alloy thin‐walled tube with a diameter of 13 mm, and a piece of acrylic plate covered with polyimide film (Figure S9, Supporting Information). The experimental results indicate that the IPCM array successfully captured and finally formed contact with these three objects after collision (Movie S8, Supporting Information).

## 3 Conclusion

In this study, an IPCM array was developed to address the overload‐induced adhesion failure that micropillar adhesives may encounter during space docking and capture. The proposed IPCM array can still maintain ≈ 85% adhesion after static large deformation, demonstrating excellent adhesion stability. The element size and layout of the IPCM array have a significant impact on its adhesion maintenance capabilities under high preloads. In addition to the design of the IPCM array given in this article, a design solution from the perspective of the non‐uniform distribution of elements can be expected. Given the possible competition between the speed of deformation recovery of micropillars after large deformation and the propagation speed of separation waves, we studied the effect of separation speed on adhesion. The results indicate that the viscoelasticity‐induced adhesion enhancement effect dominates. The finite element contact analysis of a single micropillar suggests that the large deformation of pillars during adhesion failure involves buckling and sliding behaviors, and buckling generally occurs before sliding. Moreover, it also reveals that the mechanical mechanism of the adhesive failure is the redistribution of normal stress induced by the large deformation of pillars. Finally, we conducted collision tests and compared the dynamic responses (critical incident velocity and COR) of IPCM and GM arrays to demonstrate the advantage of IPCM arrays in dynamic capture. The results indicate that the IPCM array has higher adhesion and energy dissipation in comparison with the GM array. We also demonstrated the potential of IPCM arrays in dynamically capturing different objects. The IPCM array opens ideas for the application of micropillar adhesives in space docking and capture, but there are still some aspects that require improvement and further research.

## 4 Experimental Section

### Material and Fabrication of Micropillars

The micropillar arrays were made of polypolydimethylsiloxane (Sylgard 184, Dow Corning) and prepared using the molding method. High‐temperature lamination (HTL) resin molds with hexagonally arranged microholes were fabricated using the Projection Micro Stereolithography technology (S140, BMF Precision Tech Inc.), and coated by release agent (Perfluorooctyl‐trichlorosilane) using the chemical vapor deposition process. The prepolymer base was thoroughly mixed with curing agent in a 10:1 ratio, degassed, and then poured into the HTL resin molds. The PDMS‐filled molds were degassed again until air bubbles disappeared and cured in an oven at 75 °C for 4 h. Subsequently, the PDMS micropillar arrays were carefully peeled off from the resin molds. The dimensions of the specimens prepared by the above process were characterized using a digital microscope (VHX‐7000, KEYENCE). Microscope photographs of two typical micropillar arrays are shown in Figure S1 (Supporting Information).

### Adhesion Test Apparatus

Adhesion tests were performed using a customized test apparatus, as shown in Figure S2 (Supporting Information). The backing layer of micropillar arrays was pasted on a 2 mm thick rectangular piece of transparent glass fixed on a rigid support. The rigid support was attached to a manual positioning stage (MGON series, Zolix Instruments) with 5 degrees of freedom for aligning the contacts. The probe to attach/detach the micropillar arrays is a plano‐convex lens with a diameter of 83.335 mm, which was connected to a load cell (LSB200‐1 lb, FUTEK) for measuring contact force between the probe and micropillar sample. The load cell was mounted on a precise linear motorized translation stage (L509.2ASD00, Physik Instrumente) with closed‐loop control. This linear translation stage can stably output vertical displacement loads at a certain speed. The manual positioning stage and the linear translation stage were placed on an optical vibration isolation platform to minimize the impact of environmental vibrations as much as possible. A camera (MV‐CA004–10UC, HikRobot) was placed above the samples to record images of the contact area.

The dynamic contact test apparatus, as shown in Figure S8 (Supporting Information), resembles a pendulum. An aluminum alloy pendulum rod was installed on a fixed shaft and can rotate horizontally around the shaft. A glass probe was pasted on the far end of the rod as the substrate. The micropillar specimen was fixed onto a load cell (LSB200‐1 lb, FUTEK) which was mounted on a manual positioning stage to measure the contact force between the specimen and the probe. The pendulum rod can be manually controlled to collide with the micropillar specimen at a certain initial speed. The speed of the rod end was measured using a laser vibrometer (LK‐G80, KEYENCE).

### Adhesion Measurements

Since the experiment involves micropillar buckling at which the force will suddenly decrease and become non‐monotonic, the force‐controlled testing method cannot be used. In this paper, we use the displacement‐controlled method to perform the entire testing process including loading, pause, and unloading (Figure S7, Supporting Information). In the loading stage, the glass probe was pushed onto the micropillar arrays at a constant speed of 5 µm s^−1^ until the set target compression depth was reached. Then, hold the probe for 10 s to form intimate contact. Subsequently, the probe was retracted at a speed of 5 µm s^−1^ until it was completely detached. Throughout the test, the contact force between the probe and the micropillar array was measured using the load cell. The relative displacement of the contact can be obtained according to the loading/unloading speed and corresponding time length. At the same time, images and videos of the contact area were also collected. Signals were recorded using an acquisition card (USB‐6361BNC, National Instruments).

### Finite Element Analysis (FEA)

FEA was performed using the commercial software, ABAQUS, to simulate the attachment and detachment of pillars. For simplicity, a single micropillar was only considered rather than an array in the FEA (Figure S5, Supporting Information). The plano‐convex probe meshed using an 8‐node linear brick element (C3D8R) with a 70 GPa elastic modulus and a Poisson's ratio of 0.2. The PDMS micropillar was assumed to be an elastic body due to static loading/unloading and meshed with 2220 C3D8R elements with an elastic modulus of 2.5 MPa and a Poisson's ratio of 0.49.^[^ ^53^ ^]^ The bottom of the micropillar was fully constrained, and the translation of the probe along the loading/unloading direction was released. The interaction between the contact surfaces was simulated using a bilinear cohesive zone model (Figure S5, Supporting Information), which has three independent parameters, namely maximum normal stress σ_0_ =  1 MPa, contact stiffness *k_c_* =  400 MPa mm^−1^, and adhesion work.*W*_0_ = 5 × 10^−5^ mJ mm^−2^.^[^ ^48^ ^]^ The friction at the contact interface was also considered using the “Penalty” function with a friction coefficient. The contact property was set to “surface‐to‐surface contact” with the “small sliding formulation”. Furthermore, we considered the “geometric nonlinearity” for simulating the large deformation and an “exponential decay factor” of 0.0002 for improving the convergence of FEA. The “Newton iteration method” was selected to calculate the structural response. The entire FEA includes two steps: first, the probe is pressed against the micropillar until the target depth is reached, then the probe is unloaded in reverse and finally pulled off. It should be noted that after micropillars undergo large deformation, the side of micropillars will come into contact with the probe, so there are two potential contact interfaces in the finite element model.

## Conflict of Interest

The authors declare no conflict of interest.

## Author Contributions

D.L. and R.L. contributed equally to this work. D.L. and R.L. contributed to the conceptualization, methodology, and. investigation of this article; D.L. performed visualization and the writing of the original draft; R.L. and A.C. conducted finite element simulation. Supervision and review were done by K.Y., N.G., C.X., and W.Z.

## Supporting information

Supporting Information

Supporting Information

Supplemental Movie 1

Supplemental Movie 2

Supplemental Movie 3

Supplemental Movie 4

Supplemental Movie 5

Supplemental Movie 6

Supplemental Movie 7

Supplemental Movie 8

## Data Availability

The data that support the findings of this study are available in the supplementary material of this article.
