# Supplementary material for: In‐Plane Combination of Micropillars with Distinct Aspect Ratios to Resist Overload‐Induced Adhesion Failure
Source: Adv Sci (Weinh). 2024 May 8;11(28):2400972. doi: 10.1002/advs.202400972 (PMC11267270; doi:10.1002/advs.202400972)
Supplement: Supplementary file 1 — Supporting Information [file ADVS-11-2400972-s002.pdf]

ADVANCED SCIENCE
Open Access

Supporting Information

for *Adv. Sci.*, DOI 10.1002/advs.202400972

In-Plane Combination of Micropillars with Distinct Aspect Ratios to Resist Overload-Induced Adhesion Failure

Dongwu Li, Ruozhang Li, Kangbo Yuan, Ao Chen, Ning Guo, Chao Xu* and Wenming Zhang**

Supporting Information

In-plane combination of micropillars with distinct aspect ratios to resist overload-induced adhesion failure

Dongwu Li[1], Ruozhang Li[2], Kangbo Yuan[3], Ao Chen[2], Ning Guo[1], Chao Xu[1, *], Wenming Zhang[2, *]

[1] School of Astronautics, Northwestern Polytechnical University, Xi'an 710072, China

[2] State Key Laboratory of Mechanical System and Vibration, School of Mechanical Engineering, Shanghai Jiao Tong University Shanghai, 200240, China

[3] School of Mechanics, Civil Engineering and Architecture, Northwestern Polytechnical University, Xi'an 710072, China

Correspondence to:

Chao Xu (chao_xu@nwpu.edu.cn)

Wenming Zhang (wenmingz@sjtu.edu.cn)

Contents

Figure S1 2

Figure S2 3

Figure S3 3

Figure S4 4

Figure S5 5

Figure S6 5

Figure S7 6

Figure S8 6

Figure S9 7

Table S1 8

Table S2 9

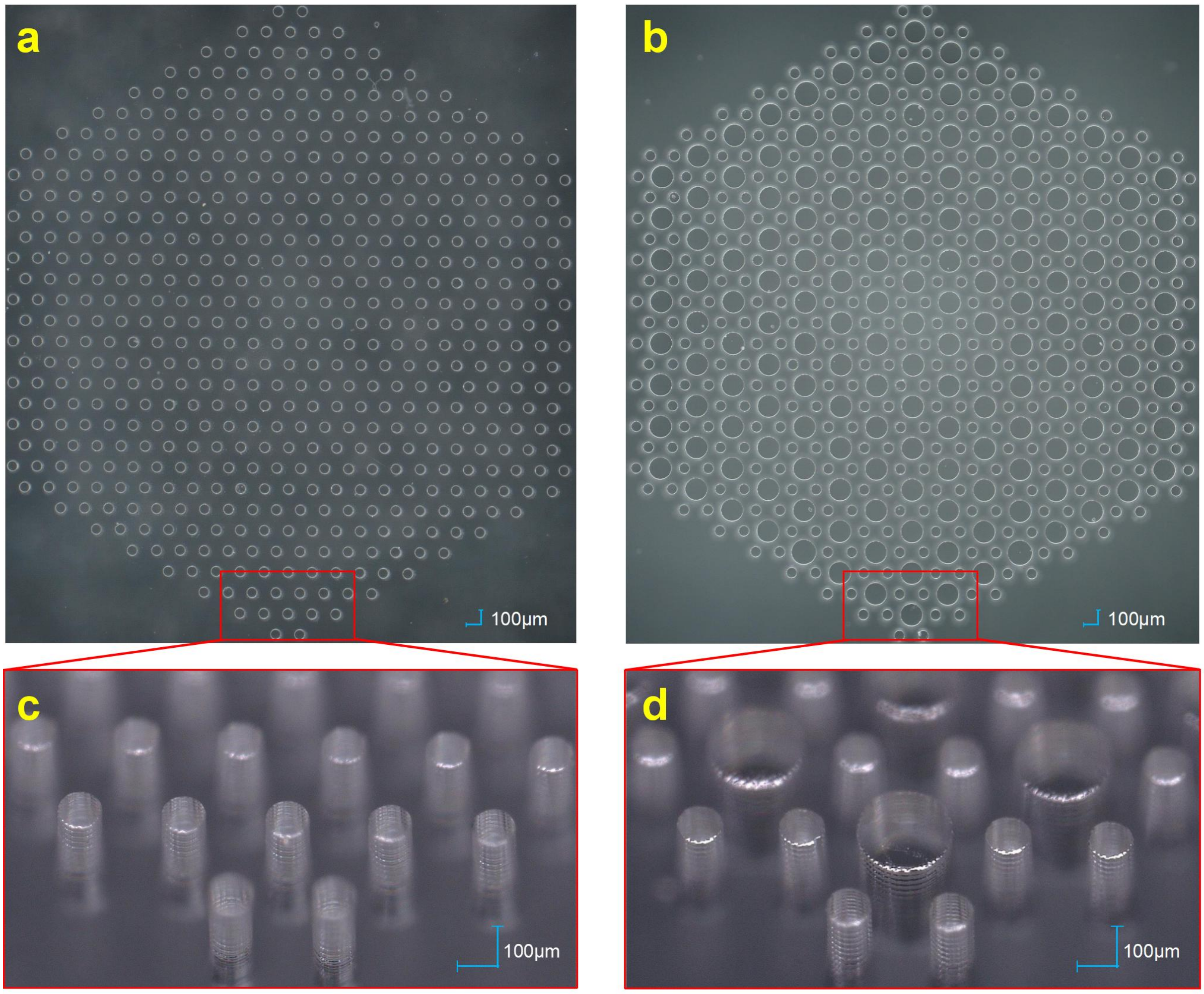

Figure S1. (a) Optical microscopic top view photo of a general micropillar array sample, (b) optical microscopic top view photo of an in-plane combined micropillar (IPCM) array sample, (c) partial side view of the general micropillar array in (a), each large micropillar has an aspect ratio of 3 (with a diameter of 100 μm), (d) partial side view of the IPCM array in (b), the aspect ratio of each large micropillar is 2 (with a diameter of 200 μm) and that of each small micropillar is 3 (with a diameter of 100 μm).

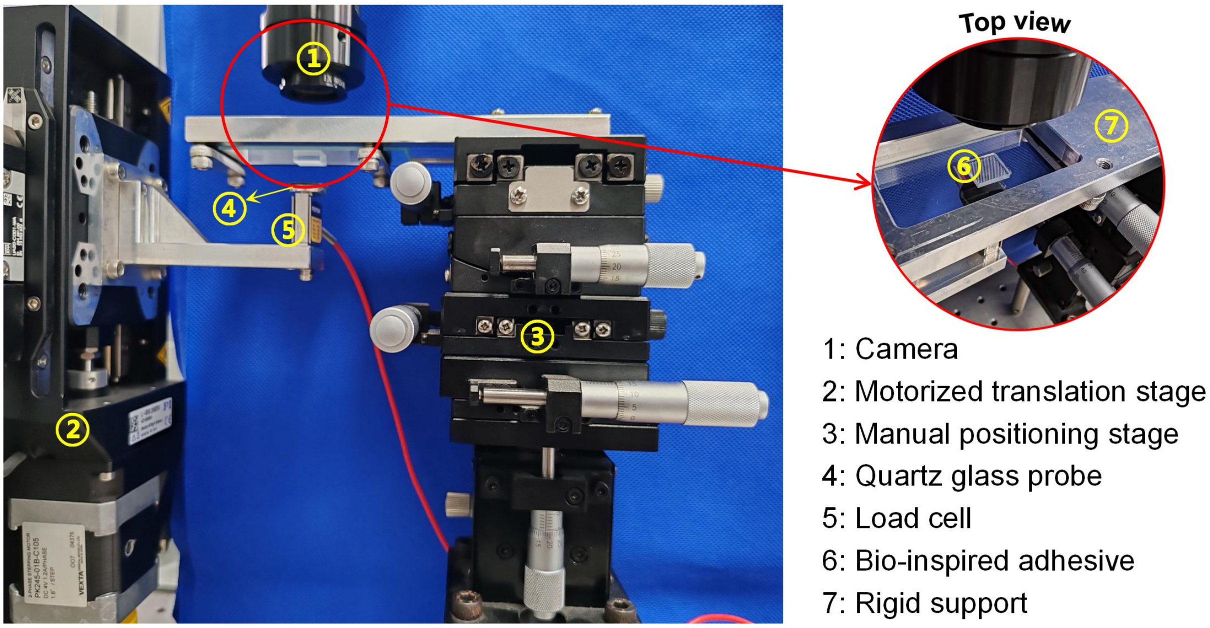

Figure S2. Photograph of customized test apparatus for adhesion measurement. The manual positioning state and the motorized translation stage are separately placed on an optical vibration isolation platform. A precise linear translation stage with closed-loop control is used to provide compressive and tensile loads. A camera is fixed above the test sample for capturing the contact images. The contact force and displacement on the adhesive surface are measured using a load cell and an built-in displacement sensor.

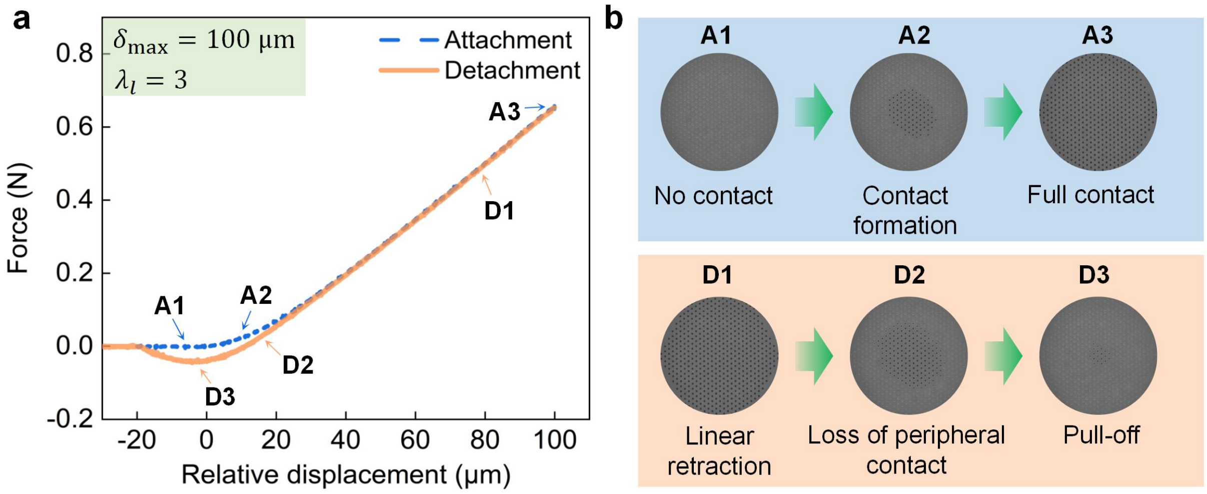

Figure S3. (a) Measured force-displacement relation of the IPCM array sample under a compression depth of 100 μm in which case no bending deformation of micropillars is induced during the entire loading and unloading process. (b) In situ pictures of the contact area between micropillars and rigid

probe at different loading and unloading moments. The dark and light areas represent the contact and detached area, respectively. The capital letters A and D represent the attachment and detachment process, respectively.

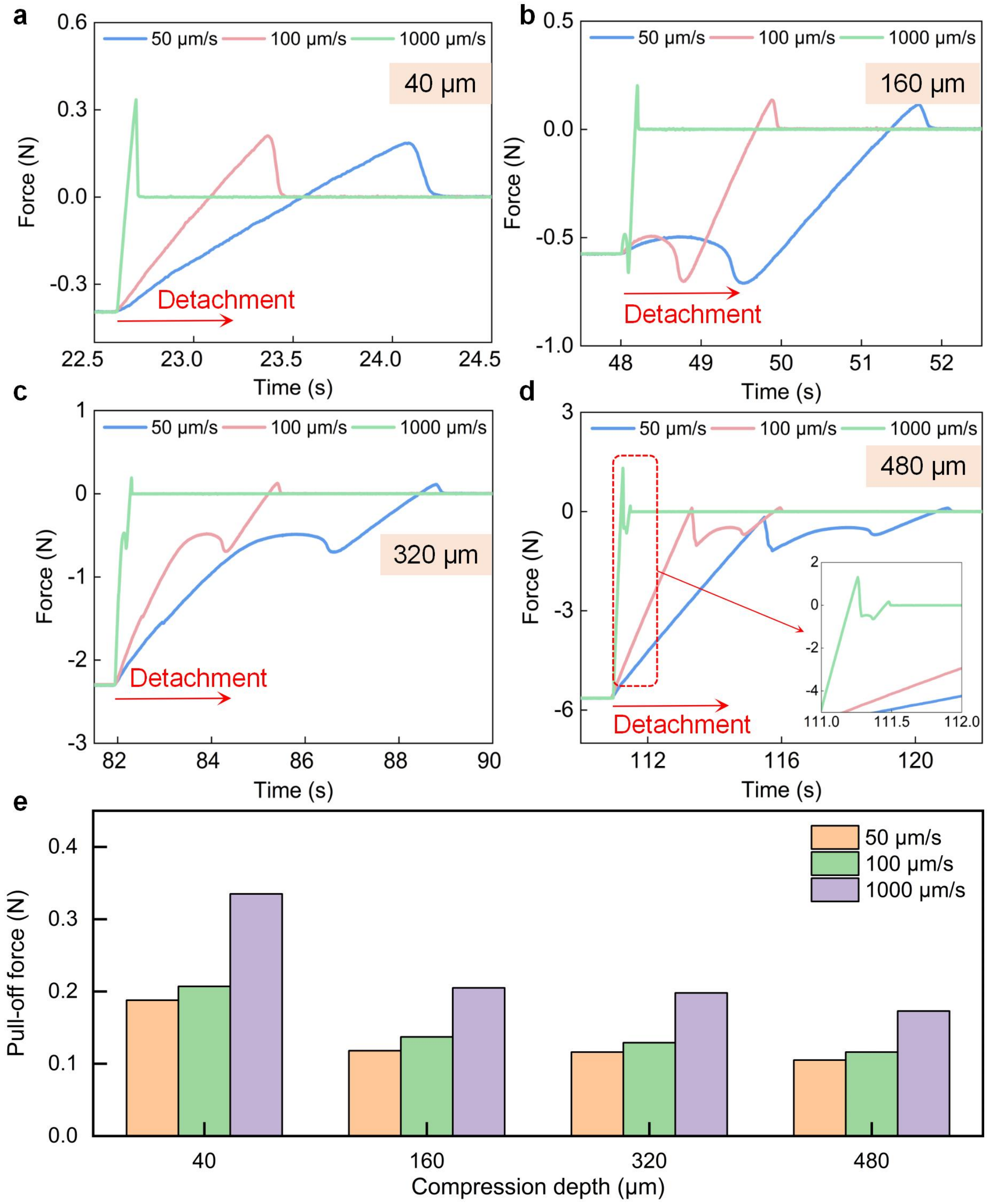

Figure S4. Force as a function of time at different detachment velocities, 50 μm/s, 100 μm/s, 1000 μm/s and different compression depths (a) 40 μm, (b) 160 μm, (c) 320 μm, (d) 480 μm. (e) Pull-off force at different detachment velocities and different compression depths. It can be seen that the compression depth (or preload) does not affect the rate-dependent adhesion.

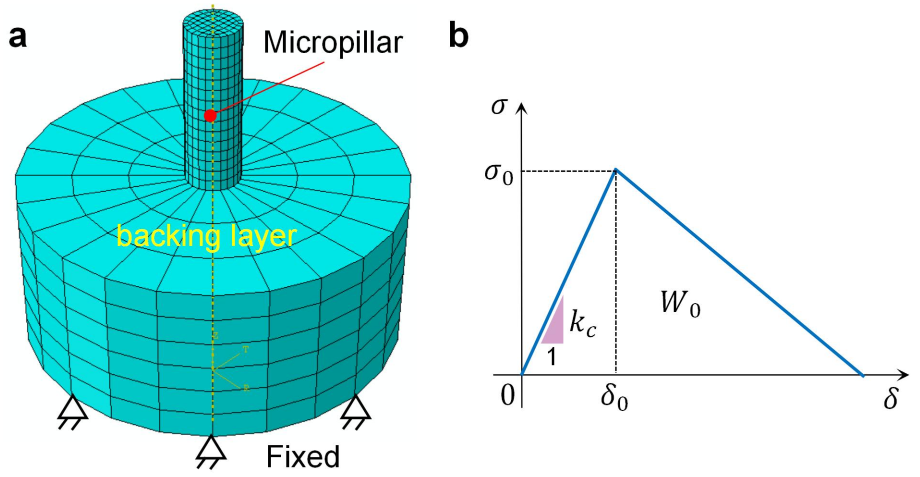

Figure S5. (a) Finite element model of a single micropillar meshed using 8-node linear brick element with a 2.5 MPa elastic modulus and a Poisson's ratio of 0.49, (b) bilinear cohesive zone model for simulating the adhesion interactions between micropillars and the probe.

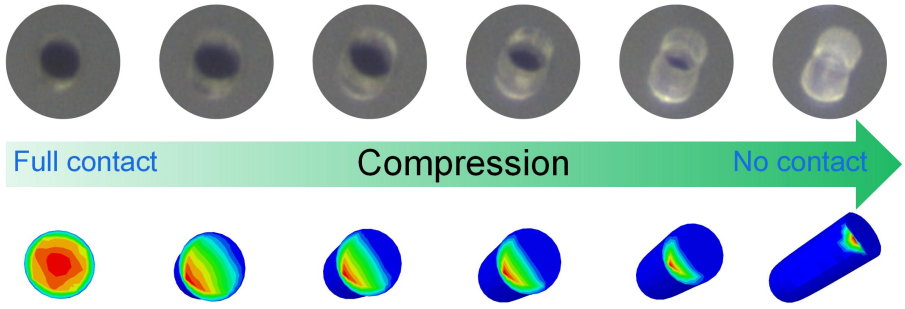

Figure S6. Comparison of the experimental contact images of a micropillar against the contact contour obtained from finite element contact analysis under monotonically increasing compressive load. As the compressive load increases, the contact state gradually changes from 'full contact' (top contact) to 'no contact' (side contact). This qualitative comparison verifies the accuracy of the finite element analysis model.

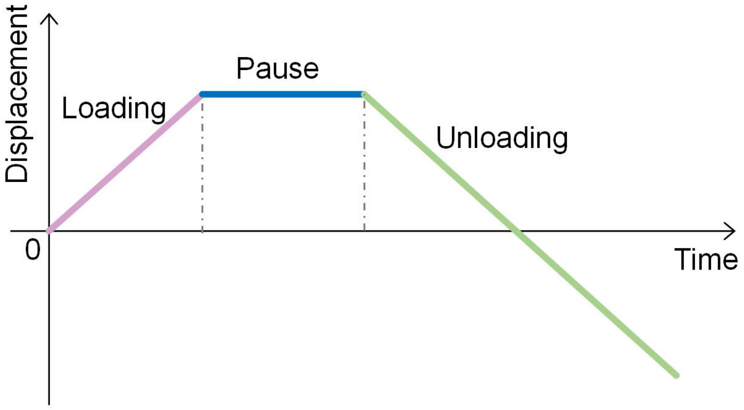

Figure S7. Illustration of the displacement-controlled testing method including three stages, namely loading, pause, and unloading. During the loading and unloading stages, the displacement increases and decreases monotonically at a constant speed, respectively. The pause phase of all tests lasted 10 seconds.

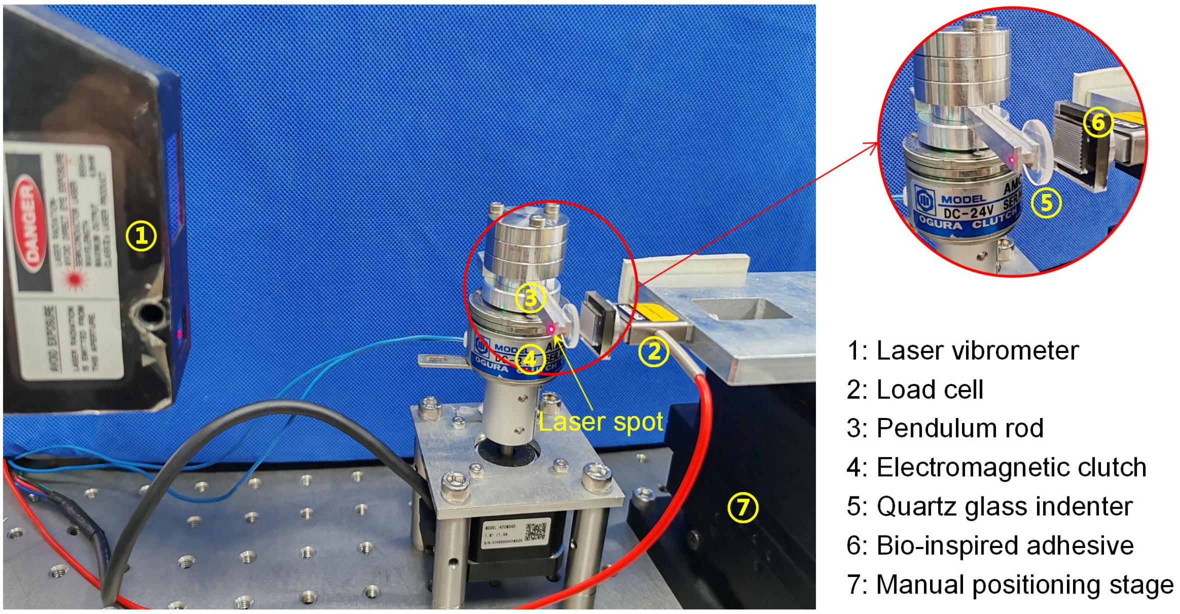

Figure S8. Photograph of the test setup simulating dynamic capture. A pendulum rod can be manually controlled to collide with the bio-inspired adhesive at a certain initial speed. The speed of the rod and contact force are measured using a laser vibrometer and a load cell.

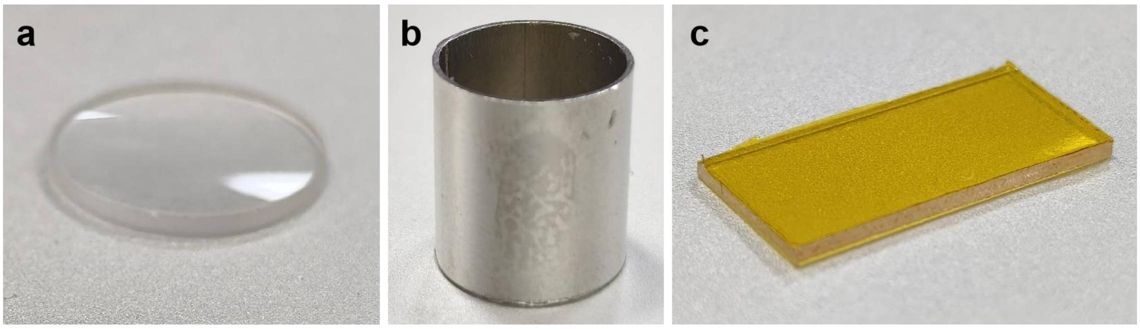

Figure S9. Three different target objects in the dynamic capture demonstration test: (a) a plano-convex lens with 83.3 mm diameter, (b) an aluminum alloy thin-walled tube with a diameter of 13 mm, (c) a piece of acrylic plate covered with polyimide film.

Table S1. Characteristic parameters of all tested samples. The symbols h and λ denote the height and aspect ratio of pillars, respectively. The area proportion is defined as the proportion of the micropillar area in the total platform area, and the volume proportion is defined as the proportion of micropillar volume in the total space.

**Sample Num.**	**Array layout, r_n**	**Pillar quantity**		**Pillar geometries (mm)**		**Area proportion**	**Volume proportion**
		Small pillar	Large pillar	Small pillar	Large pillar		
#1 (Control)	2	553	0	h_s=0.3, λ_s=3		4.3%	0.57%
#2		384	169		h_l=0.3, λ_l=2	6.0%	0.78%
#3		384	169		h_l=0.3, λ_l=1.5	8.3%	1.09%
#4		384	169		h_l=0.28, λ_l=1.4	8.3%	1.04%
#5		384	169		h_l=0.26, λ_l=1.3	8.3%	1.00%
#6 (Control)	6	657	0	h_s=0.3, λ_s=3		5.0%	0.67%
#7		546	91		h_l=0.3, λ_l=2	5.9%	0.77%
#8		546	91		h_l=0.3, λ_l=1.5	7.1%	0.93%
#9		546	91		h_l=0.28, λ_l=1.4	7.1%	0.91%
#10		546	91		h_l=0.26, λ_l=1.3	7.1%	0.87%

Table S2. Summary of pressure-sensitive micropillar adhesives and micropillar stability.

Ref.	Probe	Dimensions (μm), (diameter, height)	Preload (pressure) or displacement	Pull-off force or strength	Stability	Mechanism
[21]	flat	620, 1800	1.1 mm	0.18 N	×	Preload-controlled switchable adhesion
[43]	flat	10, 30	0.75 N	60 kPa	×	Pressure-actuated adhesive
[44]	flat	14, 33	0.1 MPa	70 kPa	×	Buckling and slip
[S1]	flat	3000, 6400	300 mN	~135 mN	✓	Composite (a stiff core and a compliant shell)
[25]	flat	100, 400	120 μm	17 kPa	×	Tunable adhesion by geometric design
[S2]	curved	50, 60	5 mN	84 kPa	✓(Stability under cyclic loading)	Micropillar with Radial gradient modulus
[S3]	flat	5, 15	1 kPa	114 kPa	✓(Stability under cyclic loading)	Composite (core-shell micropillar)
This work	curved	100/150, 300	580 μm	80 mN	✓	Resist adhesion failure by geometric design

[S1] Minsky H K, Turner K T. Achieving enhanced and tunable adhesion via composite posts. Applied Physics Letters, 2015, 106(20): 201604.

[S2] Zhu B, Tan D, Xiao K, et al. Micropillar with Radial Gradient Modulus Enables Robust Adhesion and Friction. Small, 2024: 2310887.

[S3] Bae W G, Kwak M K, Jeong H E, et al. Fabrication and analysis of enforced dry adhesives with core–shell micropillars. Soft Matter, 2013, 9(5): 1422-1427.
