# Supplementary material for: In‐Plane Combination of Micropillars with Distinct Aspect Ratios to Resist Overload‐Induced Adhesion Failure
Source: Adv Sci (Weinh). 2024 May 8;11(28):2400972. doi: 10.1002/advs.202400972 (PMC11267270; doi:10.1002/advs.202400972)
Supplement: Supplementary file 2 — Supporting Information [file ADVS-11-2400972-s005.docx]

Images of the IPCM arrays, adhesion test apparatus, exemplary force-displacement curve of IPCM array under small compression depth, effect of high detachment velocity on adhesion, finite element model and the bilinear cohesive model, evolution of top contact of a single pillar, and illustrate of the displacement-controlled loading-pause-unloading test (PDF)

Video showing the deformation of GM array at a compression depth of 240 µm (Movie S1)

Video showing the deformation of GM array at a compression depth of 500 µm (Movie S2)

Video showing the deformation of IPCM array at a compression depth of 200 µm (Movie S3)

Video showing the deformation of IPCM array at a compression depth of 300 µm (Movie S4)

Video showing the deformation of IPCM array at a compression depth of 580 µm (Movie S5)

Video showing the deformation of a single micropillar in the FE model at a compression depth of 80 µm (Movie S6)

Video showing two typical motion modes after collision (Movie S7)

Video showing demonstration tests of IPCM array dynamic capturing different target objects (Movie S8)
